# Utilization of screening for cerebrotendinous xanthomatosis in adult patients with idiopathic ataxia

**DOI:** 10.3389/fneur.2026.1763277

**Published:** 2026-03-24

**Authors:** Rebeka Sipma, Marissa Dean, Andrea DeBarber, Talene Yacoubian

**Affiliations:** 1 Department of Neurology, University of Alabama at Birmingham, Birmingham, AL, United States; 2 Department of Chemical Physiology and Biochemistry, Oregon Health and Science University, Portland, OR, United States; 3 Department of Molecular and Medical Genetics, Oregon Health and Science University, Portland, OR, United States

**Keywords:** 7α, 12α-dihydroxy-4-cholesten-3-one, adult-onset ataxia, cerebrotendinous xanthomatosis, CTX, idiopathic ataxia

## Abstract

**Introduction:**

Cerebrotendinous xanthomatosis (CTX) is an autosomal recessive lipid storage disorder. Early diagnosis and bile acid replacement therapy can improve prognosis. While clinical presentation typically occurs at a young age, recent data suggests patients presenting as adults may predominately have neurological symptoms, including ataxia. CTX prevalence among adults with idiopathic ataxia is unknown.

**Methods:**

In this study, we examined the prevalence of CTX among adult patients with idiopathic ataxia referred to a specialized ataxia clinic. We used serum blood 7α,12α-dihydroxy-4-cholesten-3-one to screen for CTX in adults presenting to our tertiary care ataxia clinic with progressive cerebellar ataxia of unknown etiology. Clinical assessment included the Scale for Assessment and Rating of Ataxia (SARA) and the CTX suspicion index score. 51 participants enrolled, and 50 samples were able to be analyzed.

**Results:**

All 50 usable blood samples showed normal 7α,12α-dihydroxy-4-cholesten-3-one levels.

**Discussion:**

CTX is a very rare cause of progressive cerebellar ataxia in a tertiary care adult ataxia clinic.

## 1 Introduction

Cerebrotendinous xanthomatosis (CTX) is a rare autosomal recessive disorder due to mutations in the *CYP27A1* gene resulting in abnormal lipid storage, which causes a variety of neurologic and systemic symptoms. *CYP27A1* encodes a mitochondrial cytochrome P450 enzyme called sterol 27-hydroxylase which catalyzes several steps in the oxidation of cholesterol into bile acids via sterol intermediates (1). This dysfunction leads to a decrease of chenodeoxycholic acid and an increase in bile acid precursors, including 7α,12α-dihydroxy-4-cholesten-3-one, and derived cholestanol and bile alcohols (1–4). These metabolites deposit diffusely in tissues but are particularly enriched in tendons, the lenses of eyes, and neuronal tissue (1). The typical phenotype of patients with CTX is marked by chronic diarrhea, bilateral juvenile cataracts, tendon xanthomas, and/or neurological symptoms typically before the third decade (5–7). Treatment with Chenodeoxycholic acid (CDCA) replacement improves the severity of symptoms and slows disease progression (8, 9). Treatment before the age of 24 was more likely to result in complete resolution of neurologic symptoms (10).

The prevalence of CTX in the USA is likely low, with an estimate of 1:72,000 to 1:150,000 (1, 11). With an estimated 336 million population in the USA in 2024 (12), one would expect ~2,000–4,000 cases of CTX. More recent prevalence estimates suggest the global prevalence may be even higher than previously reported (13). However, only several hundred cases of CTX have been reported worldwide (1). These data suggest that CTX is underreported and/or underdiagnosed and point to the importance of identifying patients who would benefit from prompt treatment to prevent disability.

CTX may present initially to a neurology clinic. Neurological features of CTX include cognitive impairment, pyramidal symptoms, epilepsy, cerebellar ataxia, parkinsonism, dystonia, myoclonus, or postural tremor (5, 14). Ataxia and/or gait disturbance was identified in 62% of patients, with a high reported rate in patients aged 30–50 years (1, 6). Recent case reports have suggested that some patients with CTX may present later in life with cerebellar ataxia (2, 8, 15–19). Given that neurological presentations for CTX can be so diverse, workups are often extensive, and diagnosis may be significantly delayed. Using the clinical suspicion index for CTX can be helpful in deciding whom to screen for CTX (6). CTX patients have elevated plasma/serum bile precursor and cholestanol levels, which can be used for screening (3, 20–22). Urine and serum can also be further analyzed for bile alcohols (1, 3, 22, 23). Gene analysis of *CYP27A1* is now available, but cost limits its utility.

In light of the case reports in which ataxia was the presenting symptoms, we examined the potential occurrence of CTX among adult patients who were referred to a specialized ataxia clinic at a tertiary care center. While genetic testing can be costly, biochemical screening for CTX is possible through measurement of blood CTX biomarker levels, such as 7α,12α-dihydroxy-4-cholesten-3-one. This additional test would be relatively easy to incorporate into preliminary screening labs for ataxia, since serum testing is usually sent as part of an initial ataxia evaluation.

## 2 Methods

### 2.1 Standard protocol approvals, registrations, and patient consents

This study was approved by the Institutional Review Board (IRB) at University of Alabama at Birmingham. Informed patient consent was obtained verbally and in writing prior to enrollment in the study. Consent was documented in writing and retained with all study documents.

### 2.2 Inclusion and exclusion criteria

**Figure 1:**
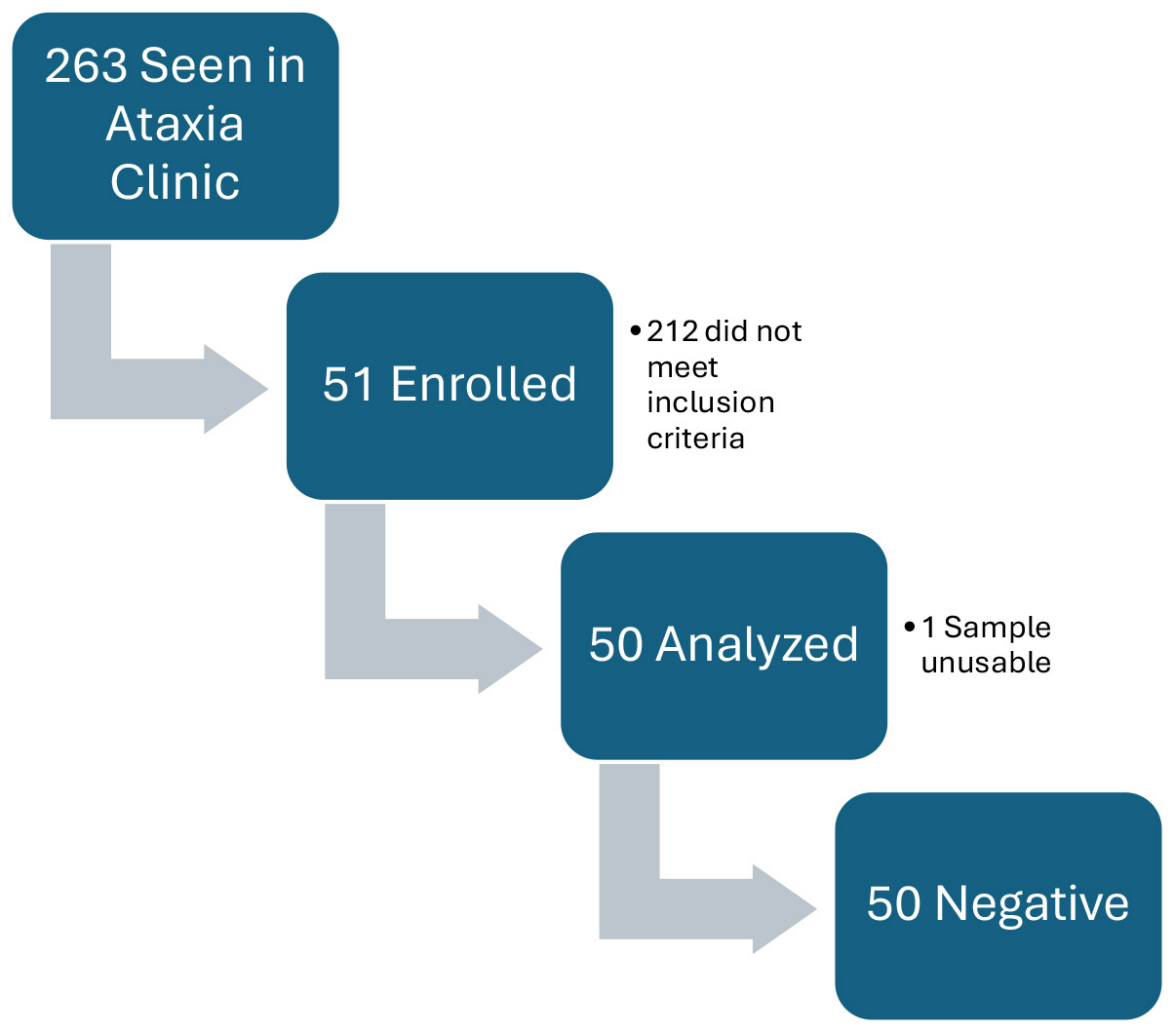
Participant flow chart.

Patients were enrolled from October 2019 to May 2024 after evaluation in The Kirklin Clinic of University of Alabama at Birmingham (UAB) ataxia clinic by one of two ataxia specialists. Enrollment goal was initially 100 based on typical clinic numbers, but during the COVID-19 pandemic, virtual visits significantly delayed enrollment requiring an extended study window and reduced enrollment goal to 50. A total number of 263 patients were seen in the ataxia during the enrollment period (Figure 1). Enrolled patients were between the ages of 18 years old and 80 years old. Inclusion criteria included a history of progressive cerebellar ataxia for at least 6 months prior to enrollment. Exclusion criteria included prior diagnosis of known genetic hereditary ataxia in the patient or family, history of documented post-stroke ataxia, and acute onset (< 24 h) of ataxia symptoms without progression after 6 months.

Subjects that were enrolled in the study previously had an initial work-up for their ataxia disorder that included brain MRI imaging and serological testing. Serological testing typically included complete metabolic panel; vitamin B1, B6, B12, and E levels; anti-glutamic acid decarboxylase 65 antibodies; anti-thyroid peroxidase antibodies; copper; zinc; selenium; ceruloplasmin; thyroid panel; gluten antibodies; and basic autoimmune panels. A considerable number of these subjects also had paraneoplastic antibody testing, cerebrospinal fluid (CSF) tests, nerve conduction studies, spinal imaging, and genetic testing for some ataxia disorders.

### 2.3 Clinical assessment

Patients who were enrolled provided demographic data, medical history, family history, results of any known prior genetic testing, and a blood sample. Additional clinical assessments included the Scale for Assessment and Rating of Ataxia (SARA) (24) and the CTX suspicion index score (6).

### 2.4 CTX screening

The blood samples were sent to Oregon Health and Science University (OHSU)'s Sterol Analysis Laboratory for measurement of the bile acid precursor 7α,12α-dihydroxy-4-cholesten-3-one. Plasma 7α,12α-dihydroxy-4-cholesten-3-one is considered more sensitive and specific to test for CTX than plasma 5α-cholestanol (22, 23). In addition to being a useful CTX biomarker in plasma, 7α,12α-dihydroxy-4-cholesten-3-one can also be measured in dried blood spots to discriminate samples from CTX affected individuals from unaffected controls (25). The majority of samples analyzed for the study were dried blood spots (48) with only two EDTA plasma samples analyzed. Published normal reference ranges and screening cut-off values were similar for dried blood spots and plasma matrices. For plasma, normal 7α,12α-dihydroxy-4-cholesten-3-one is < 10 ng/ml (22) and for dried bloodspots normal 7α,12α-dihydroxy-4-cholesten-3-one is < 25 ng/ml (25). If any samples were positive for 7α,12α-dihydroxy-4-cholesten-3-one, they would reflex to gene analysis.

### 2.5 Data availability

Anonymized data not published within this article will be made available by request from qualified investigators.

## 3 Results

### 3.1 Demographic and medical history

During the enrollment period, 51 patients met criteria and were enrolled. Of the 51 participants, 47% were male and 80% were of European ancestry with additional demographic details in Table 1. Education level ranged from seventh grade to completion of a professional degree (Table 1). The most common co-morbidities included hypertension (51%) and hyperlipidemia (39%) (Table 1). Average age at time of enrollment was 58.4 years old (range 35–80).

**Table 1:** Demographic information and medical history.

**Gender**	***n***	**Percentage**
Male	24	47.1%
Female	27	52.9%
**Ethnicity**	***n***	**Percentage**
Not Hispanic or Latino	51	100
**Race**	***n***	**Percentage**
European ancestry	41	80.4%
Black or African American	9	17.6%
Asian	1	2.0%
**Education level**	***n***	**Percentage**
7th grade	1	1.9%
8th grade	1	1.9%
9th grade	2	3.9%
11th grade	2	3.9%
12th grade, no diploma	1	1.9%
12th grade, received diploma	4	7.8%
Some college, no degree	4	7.8%
Associate degree in occupational, technical or vocational program	5	7.8%
Associate degree in academic program	7	13.7%
Bachelor's degree	13	25.5%
Master's degree	9	17.6%
Professional school degree (MD, DDS, DVM, JD)	2	3.9%
**Past medical history**	***n***	**Percentage**
Hypertension	26	51%
Hyperlipidemia/High cholesterol	20	39%
Diabetes mellitus	8	16%
Cancer	7	14%
Coronary artery disease	3	6%
Congestive heart failure	3	6%
Stroke or TIA	1	2%
Multiple sclerosis	0	0%
Dementia	0	0%

### 3.2 Family history

Ten participants identified a family history of ataxia. Four of the ten had only one first-degree relative with ataxia. One participant had one second-degree and one third-degree relative with similar symptoms. The other five had at least one first-degree relative plus 1–6 additional affected family members.

### 3.3 Ataxia symptoms

The average age of onset for ataxia symptoms was 48.8 years old (range 17–71 years). The average age at diagnosis of cerebellar ataxia was 53.8 years old (range 23–73 years). The majority (65%) were diagnosed with cerebellar ataxia within the first 5 years of symptom onset, but 8% were diagnosed more than 20 years after symptom onset. Seventeen patients reported prior genetic testing for ataxia, which was unrevealing for etiology. Eight of these subjects had undergone prior testing with the Athena Diagnostics Comprehensive or Autosomal Dominant Ataxia genetic panels only. Two subjects had prior testing with the GeneDx Xpanded Ataxia panel only. Three subjects had multiple genetic panels, including both Athena Diagnostics and GeneDx genetic ataxia panels. One subject had genetic testing for SCA6. Details of genetic ataxia testing for three subjects were unknown. Average SARA score at the time of enrollment was 15.1 (range 5–26.5). On the CTX suspicion index scale (Table 2), all participants received 50 points for presence of ataxia which is one of the “strong indicators”. Fifteen participants had only 50 points for ataxia. Twenty-six participants had polyneuropathy which was the second most common indicator observed and considered a “moderate” indicator. Three subjects had xanthomas which is considered a “very strong” indicator and given a score of 100. One participant had juvenile cataracts and 12 reported intellectual disability and/or psychiatric disturbances which are other “strong indicators.” Eight participants had parkinsonism, and one had epilepsy which were “moderate” indicators. The average score was 85.7 (range 50–175).

**Table 2:** CTX suspicion index score.

**Score**	***n***	**%**
1–50	15	29.5%
51–75	17	33.3%
76–100	7	13.7%
101–125	7	13.7%
126–150	4	7.8%
151–175	1	2.0%

### 3.4 7α,12α-Dihydroxy-4-cholesten-3-one testing

Of the 50 usable blood samples, all were within the normal range for 7α,12α-dihydroxy-4-cholesten-3-one, which was measured utilizing high performance liquid chromatography-tandem mass spectrometry (22, 23). One sample was not able to be run, and the participant declined to return for another sample to be drawn during the time of the study. Participant's data was included in all analyses apart from the sterol testing results. If screening results would have been abnormal, then genetic testing would also have been done, but none of our samples reached that threshold.

## 4 Discussion

In this study, we performed CTX blood screening in 51 adult subjects with idiopathic cerebellar ataxia who presented to the ataxia clinic at UAB. Of the 51 screened, 50 were negative for CTX. The remaining sample was not able to be processed in the laboratory due to protocol changes, and the subject declined to provide a second sample. Our findings suggest that the occurrence of CTX in a tertiary care adult movement disorders clinic in the southeastern United States is very rare. We found 0 subjects out of 50 subjects to be positive for CTX using blood testing (Clopper-Pearson 95% CI: 0 to 0.07112174). The small sample size and case-mix prevent a true measurement of prevalence.

Several limitations of our study include the small sample size, referral bias inherent to a tertiary clinic in an academic center, potential limitations of our blood screening methodology, one missing biosample, and the heterogeneity of so-called “idiopathic” ataxia. Causes of cerebellar ataxia are notoriously numerous and broad, making it a diagnostic and financial challenge to identify causes of cerebellar ataxia. Clinically, subjects showed heterogeneity in terms of the extent of previous diagnostic testing and other neurological symptoms besides cerebellar ataxia. All the subjects in this study had undergone brain imaging and serological testing at a minimum as part of the work-up for the etiology of their ataxia. Some subjects had testing limited to brain MRI and serological testing to rule out metabolic, vitamin deficiency, and autoimmune causes. Other subjects more extensive testing including paraneoplastic antibody testing, CSF tests, nerve conduction studies, and genetic ataxia testing prior to enrollment. By definition, our cohort of “idiopathic” ataxia was not homogeneous. Our intent was to determine the utility of CTX screening among people with cerebellar ataxia of unknown origin.

We used 7α,12α-dihydroxy-4-cholesten-3-one to screen blood samples for CTX in our cohort. This sterol is highly elevated in the plasma and CSF of CTX subjects (3, 4). 7α,12α-dihydroxy-4-cholesten-3-one testing is considered more sensitive and specific than plasma 5α-cholestanol (22, 23). 5α-Cholestanol can be elevated in liver disorders (26), while 7α,12α-dihydroxy-4-cholesten-3-one is considered more specific to CTX (3, 4, 22). No specific disorders or treatments have been reported to affect 7α,12α-dihydroxy-4-cholesten-3-one levels, yet it is conceivable that certain disorders or medications could possibly affect our findings. It is also possible that shipping factors could have affected our blood samples such that sterol measurements were less accurate, yet the OHSU Laboratory has performed stability studies showing that the measurement of this sterol is stable in blood spots stored up to 3 weeks. All blood samples were sent out the next business day to OHSU by overnight shipping. Of note, due to a change in the OHSU laboratory procedure, the last two blood samples that were analyzed were in the form of EDTA plasma instead of as dried blood spots. While it is theoretically possible that this change in sample could have impacted our findings, all study samples tested provided clearly negative results well below the range found for CTX subjects.

Another key limitation of our study is the potential applicability of our findings to other patient populations. Genetic data in the Genome Aggregation Database (gnomAD) alongside the Human Gene Mutation Database (HGMD), the ClinVar database, and VarSome variant classifier were recently used to estimate prevalence of pathogenic *CYP27A1* variants and carrier frequency in different populations using the Hardy-Weinberg principle (13). They found East Asian populations to have highest estimated prevalence at 1 per 44,407 to 71,089 and South Asian populations close behind at 1 per 93,084 to 105,299. European and African estimates were 1 per 233,597 to 393,497 and 1 per 166,440 to 472,468, respectively. Our patient population at UAB consists primarily of patients who report European and/or African heritage. Our study enrolled only one Asian participant and no Hispanic or Latino patients. Further studies would need to be done to determine if screening for CTX would be more useful in clinics with higher proportions of Asian and Hispanic or Latino patients.

The authors of the clinical suspicion index for CTX suggested a score ≥ 100 warranted serum testing (6). In our study, patients would score no less than 50 points as presence of ataxia was part of the inclusion criteria. Only 37% scored ≥ 100, and none scored ≥ 200. Using the suspicion index to screen patients in movement disorder clinic could be useful for determining when to send blood for screening for CTX.

In conclusion, while blood CTX biomarker levels are a minimally invasive method of testing for this treatable disease, CTX is likely a very rare cause of cerebellar ataxia among adults. If clinicians have a high index of suspicion for CTX, then biochemical screening should still be considered. Dried blood spot screening provides easy sample collection and shipping and is relatively inexpensive compared to genetic testing.

## Data Availability

The raw data supporting the conclusions of this article will be made available by the authors, without undue reservation.
